# Systematic Review and Meta-Analysis of the Sero-Epidemiological Association between Epstein Barr Virus and Multiple Sclerosis

**DOI:** 10.1371/journal.pone.0061110

**Published:** 2013-04-09

**Authors:** Yahya H. Almohmeed, Alison Avenell, Lorna Aucott, Mark A Vickers

**Affiliations:** 1 Health Services Research Unit, University of Aberdeen, Foresterhill, Aberdeen, United Kingdom; 2 School of Medicine and Dentistry, University of Aberdeen, Aberdeen, United Kingdom; 3 Division of Applied Medicine, University of Aberdeen, Aberdeen, United Kingdom; United Arab Emirates University, United Arab Emirates

## Abstract

**Background:**

A role for Epstein Barr virus (EBV) in multiple sclerosis (MS) has been postulated. Previous systematic reviews found higher prevalences of anti-EBV antibodies in MS patients compared to controls, but many studies have since been published, and there is a need to apply more rigorous systematic review methods.

**Methodology/Principal Findings:**

We examined the link between EBV and MS by conducting a systematic review and meta-analysis of case-control and cohort studies that examined the prevalence of anti-EBV antibodies in the serum of cases and controls. We searched Medline and Embase databases from 1960 to 2012, with no language restriction. The Mantel-Haenszel odds ratios (OR) for anti-EBV antibodies sero-positivity were calculated, and meta-analysis conducted. Quality assessment was performed using a modified version of the Newcastle Ottawa scale. Thirty-nine studies were included. Quality assessment found most studies reported acceptable selection and comparability of cases and controls. However the majority had poor reporting of ascertainment of exposure. Most studies found a higher sero-prevalence of anti-EBNA IgG and anti-VCA IgG in cases compared to controls. The results for anti-EA IgG were mixed with only half the studies finding a higher sero-prevalence in cases. The meta-analysis showed a significant OR for sero-positivity to anti-EBNA IgG and anti-VCA IgG in MS cases (4.5 [95% confidence interval (CI) 3.3 to 6.6, p<0.00001] and 4.5 [95% CI 2.8 to 7.2, p<0.00001] respectively). However, funnel plot examination suggested publication bias for the reporting of the anti-EBNA IgG. No significant difference in the OR for sero-positivity to anti-EA IgG was found (1.4 [95% CI 0.9 to 2.1, p = 0.09]).

**Conclusion/Significance:**

These findings support previous systematic reviews, however publication bias cannot be excluded. The methodological conduct of studies could be improved, particularly with regard to reporting and conduct of laboratory analyses.

## Introduction

Multiple Sclerosis (MS) is a complex, chronic, inflammatory, neurological disease that affects the central nervous system [1]. Worldwide there are 2.5 million people with MS [2] and in the United Kingdom (UK) alone the number is estimated to be 100,000 people [3]. The public health burden is huge and related to both direct medical care and loss of productivity from disability that the disease causes. It was estimated that the overall cost of MS in the UK was £1.5 billion in 1999 and a recent study estimated the mean annual cost to be over £30,000 pounds per patient [4].

The aetiology of the disease is still not well understood. Both genetic and environmental factors play roles in the development of the disease [5]. Environmental factors have been an area of intense research lately, particularly into infectious agents that could be linked to MS and many bacterial and viral agents have been studied [6]

Of all infectious agents, Epstein Barr virus (EBV) has been most strongly associated with MS [7].

The odds ratio (OR) for MS patients to be EBV sero-negative was found to be 0.06 in a review by Ascherio and Munger [7], and in a more recent meta-analysis combining results from 22 studies the OR was found to be 0.18 [8]. It also appears that the titres of anti-EBV antibodies are significantly higher among sero-positive MS cases when compared with sero-positive controls. Prospective studies suggest this increase in anti EBV antibodies is apparent from 5 to 20 years before the onset of MS [9].

The most recent systematic review that updated the association between MS and sero-positivity for different anti-EBV antibodies was that of Santiago *et al*. from 2010 [10]. Literature search terms were limited and only publications in English and Spanish were included [10]. This increases the likelihood of publication bias affecting the results. Many studies have been published more recently, which could help to estimate the association more fully. Therefore, we undertook a systematic review and meta-analysis with a wider, more comprehensive search strategy, with no language restriction and including both older and more recently published studies.

## Materials and Methods

The methods of the systematic review followed a pre-specified protocol developed by the authors.

### Search strategy

Medline and Embase were electronically searched using a comprehensive list of MeSH and Emtree headings and text words that were informed by previous reviews and from search tools in Ovid Medline and Embase (Appendix S1). The search was conducted for articles published from 1960 to March week one, 2012, with no language restriction.

For a study to be included in the review, it had to be a case-control or a cohort study, recruited patients with MS diagnosis (confirmed or probable) and controls with no MS diagnosis (healthy or non-healthy). Participants could be from any age group and studies measured serum anti-EBV IgG antibodies (using any method) in both cases and controls. We had few exclusion criteria in order to be as generalisable as possible. Exclusion criteria were non-human studies, very small studies with less than 20 cases or 20 controls and studies that only measured anti-EBV IgM (indicating recent infection).

Titles and abstracts for inclusion were reviewed by one author. A second author reviewed part of the search results (140 out of 1056 reports) and a kappa statistic for the degree of agreement between the two reviewers was calculated and was high at 0.76. Full texts of potentially relevant articles were then screened by one author and uncertainties discussed with a second author. Reference lists from included studies were hand searched to identify any further related studies. Where extra data were required, the author of the article was contacted to obtain further information. Relevant data from each study were initially extracted independently by two authors (23 out of 39 studies). After sufficient agreement was reached, data were extracted by one author and checked by a second author.

### Quality assessment

The quality assessment of included studies was based on the Newcastle-Ottawa assessment scale (NOS) [11]. We modified the exposure assessment criteria so that the subcategories would be applicable to serological studies. We added a two stars for blinding of blood sample analysts, one star for conducting the analysis in a clinical laboratory (away from investigators), one star for mentioning explicit laboratory cut-offs for sero-positivity, and one star for reporting the presence or absence of missing data.

### Data analysis

The Mantel-Haenzsel odds ratio (OR) of sero-positivity to EBV was calculated for each anti-EBV antibody (anti-Epstein-Barr Nuclear Antigen 1 (anti-EBNA1) or EBNA complex, anti-Viral Capsid Antigen (anti-VCA) and anti-Early Antigen (anti-EA)), and for sero-negativity to EBV using Review Manager Version 5.1 [12]. The ORs were combined in a meta-analysis for each anti-EBV antibody and for overall EBV sero-negativity using a pre-specified more conservative random effects model (anticipating study heterogeneity) with a 95% confidence interval (CI) for all analyses. I^2^ was used to assess heterogeneity between studies [13]. In meta-analyses with sufficient number of studies, a funnel plot [13] was inspected visually to assess for publication bias.

We conducted subgroup and sensitivity analysis testing of the meta-analyses in Review Manager version 5.1 to compare the OR of sero-positivity for the three anti-EBV antibodies in the following categories:

1. Paediatric versus (vs.) adult studies. We considered studies with median participant's age of 20 or less as paediatric,
2. Studies above median latitude vs. those below median latitude. All studies were assigned to latitude according to area of recruitment of participants. If the area of recruitment was not clear, studies were assigned to latitude according to the address of the lead author,
3. Studies controlling for age vs. those that did not,
4. Studies controlling for gender vs. those that did not,
5. Studies using McDonald[14]/Poser criteria[15] for MS diagnosis vs. those that did not or criteria used were not clear,
6. Studies that included only cases with confirmed MS diagnosis vs. those that included confirmed and probable cases,
7. Studies that used Immunofluorescence assays (IFA) vs. those which used Enzyme-linked immunosorbent assays (ELISA),
8. Studies with overall quality assessment scores above the median in the modified NOS scale compared to those scoring below the median.

To minimise the effect of multiple comparisons, we used 99% CI for all subgroup and sensitivity analyses.

## Results

**Figure 1:**
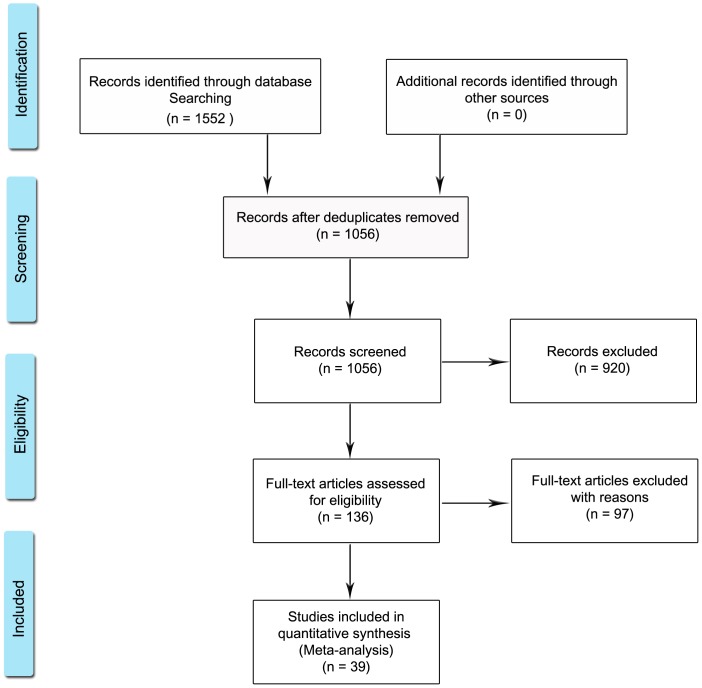
Studies identification in search strategy. Adopted from PRISMA 2009 Flow Diagram[16] (Moher, et al., 2009)^*^.

After removing duplicate reports, the search through Medline and Embase yielded 1056 reports (Figure 1, adapted from Moher, et al., 2009 [16]). We then excluded case reports, non-human studies, studies with no serological studies performed or with unclear serological results, studies and conference abstracts with no obtainable full reports, retracted studies, studies with an updated version, studies using inappropriate cases (non-MS) or with unclear case definition and studies with cases or controls less than 20. Total number of included studies was 39[17]–[55] (Table 1). We received extra unpublished data form five studies included in the review [28], [29], [35], [49], [52].

**Table 1:** Characteristics of participants in the included studies.

Study ID	Cases				Controls					
	Total	Source/Type	Sex	Age	Total	Source/Type	Sex	Age	Anti-EBV (IgG)	Test used
			F:M number and/or Ratio	Mean (SD) and/or range			F:M Number and/or ratio	Mean (SD) and/or range		
Alotaibi 2004[17]	30	MS patients enrolled in paediatric clinic	1.31	13.40 (3.63)	143	53 HC from BMT donors and 90 from ED	1.57 (ED), 0.66 (BMT)	13.37 (3.62) (ED), 10.30 (3.78) (BMT)	EBNA‡, VCA, EA	ELISA
Ascherio 2001[18]	144	New MS incidents in NHS I and II	144 All females	–	288	Healthy controls matched for Age and Cohort	288F	–	EBNA(1, 2, Complex), VCA	IFA
Banwell 2007 [19]	126	Definite MS diagnosed before the age of 18	1.5	14.1/2.2–19.6	96	HC from dental or orthopaedic procedures or emergency room	–	–	EBNA1, VCA	ELISA
Bray 1983 [20]	313	Consecutive cases with a diagnosis of MS	–	–	406	Blood donors and patients with non-demyelinating neurological diseases	–	–	VCA	IFA
Buljevac 2005 [21]	54	MS cases form Rotterdam Study	–	39.3	52	HC	–	39.3	EA	ELISA
Comabella 2010 [22]	25	24 with confirmed MS and 1 CIS patient	16: 9/1.7	36.2 (10.7)	46	Healthy full siblings	24: 22/1.1	36.3 (12.8)	EBNA1, VCA	ELISA
Ferrante 1987[23]	30	Newly diagnosed confirmed MS cases	–	–	42	21 with OND and 21 NND	–	–	EBNA‡, VCA	IFA
Gutierrez 2002 [24]	41	MS patients recruited at a regional referral centre	23: 18/1.3	39.2(12.7)	31	Patients recruited form the same centre with OND	18: 13/1.4	48.2(16.2)	VCA	ELISA
Haahr 2004 [25]	53	MS patients newly diagnosed and <33 years	49: 4/12.3	28.3/21–32	53	HC who had contact with the MS patients during childhood	49: 4/12.3	28.1/22–32	EBNA1	ELISA
Ingram 2010 [26]	75	MS patients	52: 23/2.26	39.9(11.2)	25	Subjects not related, with no history of neurological disease	11: 14/0.79	49.4 (20.6)	EBNA1	ELISA
Jafari 2010 [27]	114	61 clinically definite MS and 53 CIS	–	38 (11.5)	62	Patients with NIND	–	44 (18.6)	EBNA1	ELISA
Jaquiery 2010 [28]	40	Early MS patients	–	33 (11)	83	39 with OIND and 44 with NIND	–	OIND 44(20), NIND 45 (20)	EBNA1, VCA, EA	ELISA
Jilek 2008 [29]	73	MS and CIS patients	–	RRMS 41(7), SPMS 57(16), PPMS 55(7)*	56	35 patients with OND and 21 healthy controls	–	HC 35(10), OND 29(20)*	EBNA1, VCA, EA	ELISA
Khaki 2011 [30]	61	Newly diagnosed MS patients	52: 9/5.8	32(17)	60	Normal individuals with no signs of MS	50: 10/5	35(15)	Not specified	ELISA
Lalive 2011 [31]	22	Paediatric MS patients	18: 4/4.5	14.9(2.5)	20	7 patients with viral encephalitis and 13 HC	9: 11/0.82	Encephalitis: 7.1(6.2), HC 11(3.4)	EBNA‡, VCA, EA	ELISA
Larsen 1985 [32]	93	MS patients	44: 49/0.90	32*/17–62	93	Health controls	44: 49/0.90	32*/17–62	EBNA‡, VCA	IFA
Levin 2010 [33]	305	Confirmed or probable MS patients	103: 202/0.51	23.5(5.4)/16–40	610	Non-MS controls from same population	206: 404/0.51	23.5(5.4)/17–41	EBNA1, VCA	IFA
Lindsey2010 [34]	80	MS patients	51: 29/1.76	35.7(9.8)	80	Not clear	51: 29/1.76	34.2(11.7)	EBNA1, VCA	ELISA
Lucas 2011 [35]	206	Cases with first clinical diagnosis of CNS demyelination	–	18–59	217	Non-MS controls recruited from electoral role	–	18–59	EBNA Complex, VCA, EA	ELISA/IFA
Martyn1993 [36]	214	Clinically definite MS (105), CIS (56), acute optic neuritis (64)	–	32(7.6)	164	98 HLA DR2 positive subjects from bone marrow donors and 66 patients with OND from the same hospital	1.12	33.2(8.8)	VCA	IFA
Mowry 2011 [37]	120	MS or CIS cases with onset of disease below the age of 18	76: 44/1.7	15(3.5)	20	OND patients	12: 8/1.5	13.8(3.9)	EBNA1, VCA	ELISA
Munch 1998 [38]	138	MS patients	81: 57/1.4	43*/22–72	138	Health controls	81: 57/1.4	43*/22–72	EBNA1, EA	ELISA
Myhr 1998 [39]	144	MS patients	82: 61/1.36	39.2/17–66	170	Patients with minor gynaecological, plastic surgery disorders or traumatic fractures	93: 77/1.2	40.0/18–77	EBNA‡, VCA, EA	ELISA/IFA
Nociti 2010 [40]	267	RRMS cases	191: 76/2.5	37.7 (12)	138	50 patients with CIDP and 88 with ALS	CIDP 24: 26/0.92, ALS 42: 46/0.91	CIDP 55.3 (16.9) ALS 58.3(12.9)	EBNA1, VCA	IFA
Pohl 2006 [41]	147	MS patients	98: 49/2	13.41/4.93–20.25	147	47 healthy siblings of patients with adrenoleukodystrophy or neuroblastoma. 100 other HC from paediatric surgical clinic	98: 49/2	13.48/5.40–20.36	EBNA1, VCA, EA	ELISA/IFA
Ponsonby 2005 [42]	136	MS patients from Tasmania, Australia	92: 44/2.1	43.5 (9.3)	272	Controls recruited from the voters roll	184: 88/2.1	43.6 (9.2)	VCA	ELISA
Riverol 2007 [43]	172	MS patients	106: 66/1.6	39(9)	85	Health controls	1.6	39 (9)	EBNA‡, EA	ELISA
Sellner 2010 [44]	55	RRMS patients	38: 17/2.24	42.1 (12.7)	56	7 with OIND, 29 with NIND, 20 HC	30: 26/1.15	41.3 (14)	EBNA1	ELISA
Selter 2010 [45]	25	25 children with CIS	14: 11/1.27	9.1/2.5–15.5	58	30 HC and 28 with OND	27: 31/0.87	HC: 11.0/9–13, OND 6.0/0.3–14	EBNA1	ELISA
Shirodaria 1987 [46]	26	MS cases (Allison and Millar criteria)	17: 9/1.9	51.9/28–74	26	Healthy blood donors	17: 9/1.9	52/27–74	EBNA‡, VCA	IFA
Sumaya 1980 [47]	157	MS patients diagnosed according to Shumacher criteria	92: 65/1.48	11–>60	81	28 laboratory personnel, 33 spouses and 28 household members	45: 36/1.25	11–>60	VCA	IFA
Sumaya 1985 [48]	104	Patients with definite MS (Shumacher Criteria)	64: 40/1.6	44*/23–66	175	26 healthy sibling, 45 OND patients and 104 HC	95: 80/1.2	HC 44/20–64, Siblings:46/23–67, OND: 49/18–75	EBNA1, VCA, EA	IFA
Sundqvist 2012 [49]	585	Newly diagnosed cases of MS	–	–	664	Controls selected randomly from a national register	–	–	EBNA1	ELISA
Sundström 2004 [50]	234	MS patients	192: 42/4.6	Pro: 28/17–59,* Retro 40/19–68*	702	Controls form the same cohorts	Matched	Matched	EBNA1, VCA	ELISA/IFA
Villegas 2011 [51]	76	MS patients	62: 14/4.43	34.9(9.7)/18–59	75	Patients scheduled for minor surgery	49: 26/1.88	45.6(12.9)/20–73	EBNA1	ELISA
Villoslada 2003 [52]	98	49 RRMS patients and 49 SPMS patients	65: 33/2	RRMS: 32.1 (7.1)/18–50 SPMS: 45 (9.7)	50	Healthy blood donors	34: 16/2.1	28.7(6)/Matched with RRMS	EBNA1, EA	ELISA
Wandinger 2000 [53]	108	MS patients diagnosed according to Poser criteria	67: 41/1.6	37.9(10.3)/20–57	163	Healthy blood donors	89: 74/1.2	36.2 (11.8)/19–56	EBNA1, EA	ELISA
Waubant 2011 [54]	189	Paediatric MS patients, with disease onset before age of 18	124: 65/1.9	14.9(3.3)	66	38 OND paediatric patients, 28 healthy paediatric controls	44: 22/2	14.7(4.1)	EBNA1, VCA	ELISA
Zivadinov 2006 [55]	140	MS patients diagnosed according to Poser criteria	90: 50/1.8	42.1(10.9)	131	Healthy blood donors	1.8	Matched	VCA	ELISA

**Abbreviations: ALS**: Amyotrophic Lateral Sclerosis, **BMT**: Bone marrow transplant, **CIDP**: Chronic Inflammatory Demyelinating Polyradiculoneuropathy, **ED**: Emergency department controls, **F**: Female, **HC**: Healthy controls, **M**: Male, **NHS**: Nurses' health study, **NIND**: Non-inflammatory neurological diseases, **OIND**: Other inflammatory neurological diseases, **OND**: Other neurological diseases, **PPMS**: Primary progressive MS, **Pro**: Prospective sample, **Retro**: Retrospective sample **RRMS**: Relapsing remitting Multiple sclerosis, **SPMS**: Secondary progressive Multiple sclerosis.

* Median and inter-quartile (IQ) range provided instead of Mean and Standard deviation (SD).

‡ The Study did not specify what type of anti-EBNA IgG was measured.

The characteristics of participants in the included studies are summarised in Table 1. All studies included were either case-control studies or nested case-control in cohort studies. There were 5020 cases and 5844 controls. The median sample size of cases and controls was 108 and 82, respectively. Around half of the studies specified using McDonald or Poser criteria for MS diagnosis (21 out of 39 studies) with cases including confirmed, probable and clinically isolated syndrome (CIS) patients. Controls included healthy and non-healthy participants (patients with neurological and non-neurological diseases), with the majority of studies recruiting healthy control samples (28 out of 39). Most studies recruited hospital controls (24 out of 39). The rest either recruited samples from the community or did not state the source. There were seven paediatric studies [17], [19], [31], [37], [41], [45], [54] and 32 adult studies.

### Anti-EBNA IgG

There were 30 studies that tested for anti-EBNA IgG (anti-EBNA1 or EBNA complex IgG) sero-prevalence (for details of type of anti-EBNA IgG measured in each study, see Table 1). We divided the study by Sundstrom *et al*. [50] into two, retrospective and prospective, making the total number of studies 31. The median sero-positivity in the 31 studies in cases was 98% compared to 88% in controls.

**Figure 2:**
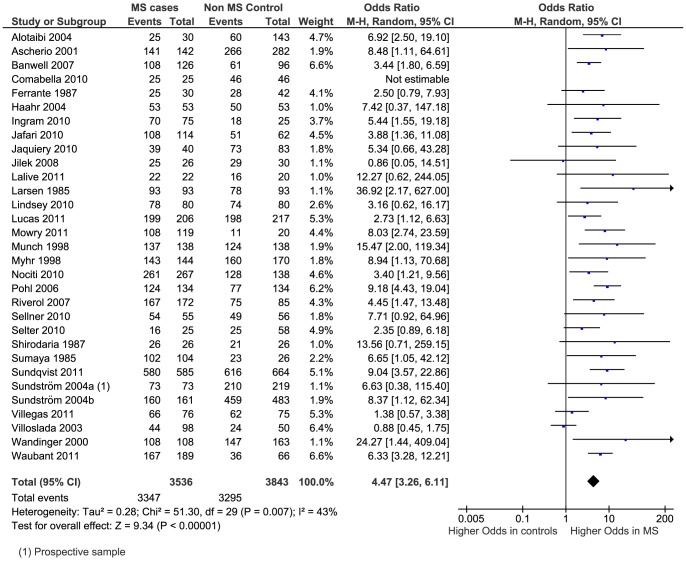
Forest plot of the Odds Ratio for anti-EBNA sero-positivity.

The meta-analysis generated an overall OR of 4.47 (95% CI 3.26–6.11, p<0.0001) (Figure 2). There was considerable heterogeneity between studies with I^2^ = 43% (p = 0.007). Removing the two studies [18], [35] which measured EBNA complex had no effect on the overall result (OR 4.56, 95% CI 3.27–6.37). Another seven studies [17], [23], [31], [32], [39], [43], [46] did not clearly state which form of EBNA was measured; if these are also removed the result is little changed (OR 4.30, 95% CI 2.90–6.39).

**Figure 3:**
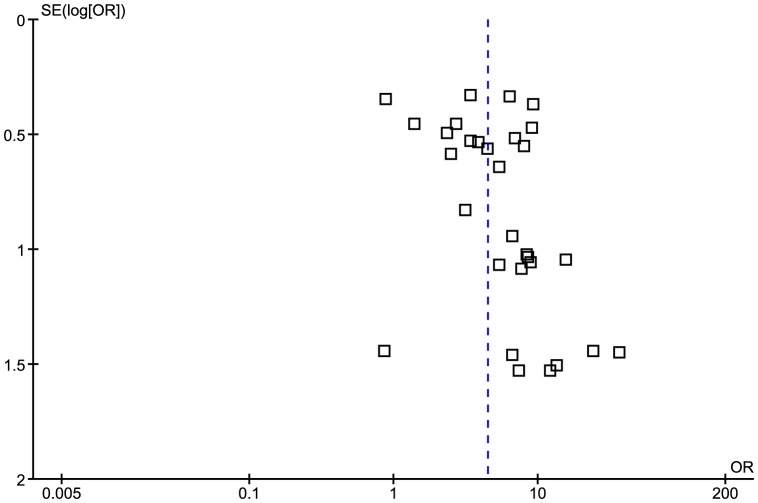
Funnel plot for studies testing for anti-EBNA IgG sero-positivity.

The funnel plot revealed an asymmetrical distribution of studies around the line of identity, indicating the possibility of publication bias (Figure 3).

### Anti-VCA IgG

There were 24 studies that tested for anti-VCA IgG sero-prevalence. After dividing the study Sundstrom *et al*. [50] into two the total number became 25. The median anti-VCA IgG sero-positivity in the 25 studies was higher in cases than controls (99% compared to 92%).

**Figure 4:**
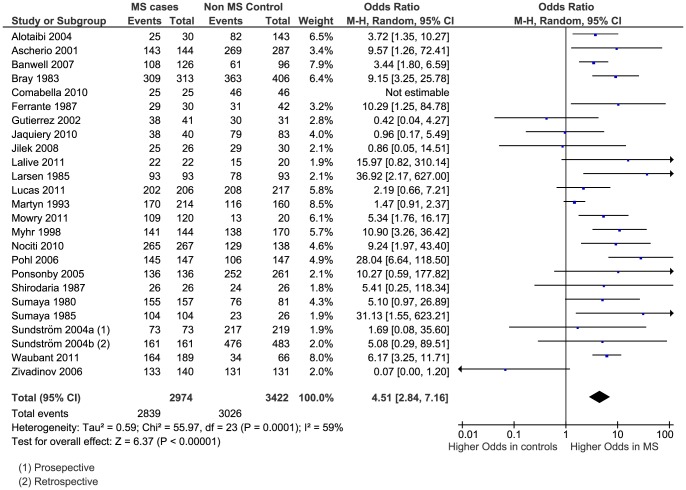
Forest plot of the Odds Ratio for anti-VCA IgG sero-positivity.

**Figure 5:**
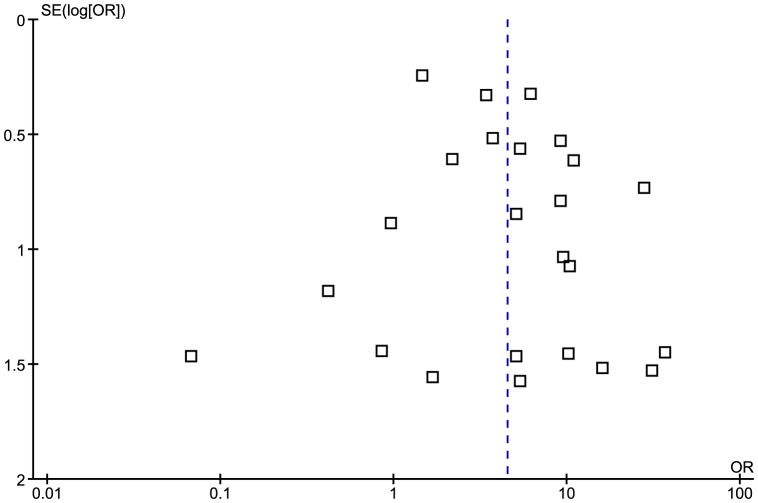
Funnel plot of studies testing for anti-VCA IgG sero-positivity.

The meta-analysis generated an OR of 4.51 (95% CI 2.84 to 7.16, p<0.00001) (Figure 4). Again there was considerable heterogeneity between studies with I^2^ = 59% (p = 0.0001). A funnel plot revealed a slightly less asymmetrical distribution of studies than for anti-EBNA IgG (Figure 5).

### Anti-EA IgG

There were 14 studies that tested for anti-EA IgG sero-prevalence. The median sero-positivities in the 14 studies in cases and controls were similar (18% and 16% respectively).

**Figure 6:**
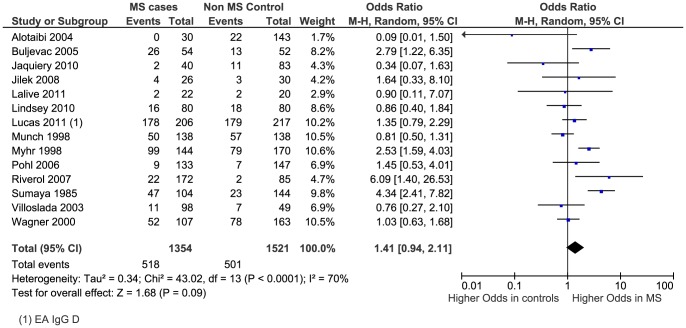
Forest plot of the Odds Ratio for anti-EA IgG sero-positivity. Note: the study Wagner, et al., (2000)[56] in Figure 6 is an older version of the study Wandinger, et al., (2000)[53].

**Figure 7:**
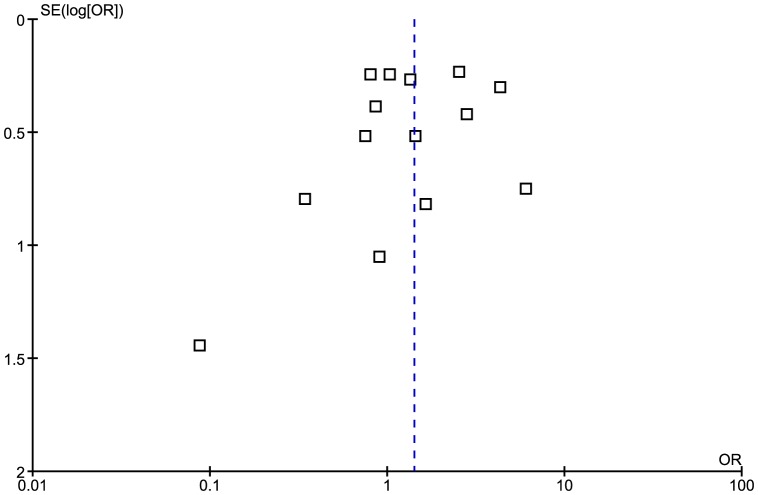
Funnel plot of studies testing for anti-EA IgG sero-positivity.

The meta-analysis generated an overall OR of 1.4 (95% CI 0.9 to 2.1, p = 0.09) (Figure 6 - Note: the study Wagner, et al., (2000)[56] in Figure 6 is an older version of the study Wandinger, et al., (2000)[53]). The heterogeneity higher than that of the previous two analyses with I^2^ = 70% (p<0.0001). The funnel plot revealed a more symmetrical distribution of studies, suggesting publication bias is less likely (Figure 7).

### Anti-EBV IgG sero-negativity

We considered sero-negativity data as adequate if the authors mentioned explicitly that cases and controls were negative for all anti-EBV anti-bodies (EBNA, VCA and EA) or mentioned that they were EBV negative without specifying which anti-EBV antibody they were negative for. Based on this, only seven studies provided data for the analysis [17], [30], [33], [38], [39], [41], [53]. The median prevalence of anti-EBV IgG sero-negativity in the seven studies was lower in cases than controls (0.7% compared to 10%).

**Figure 8:**
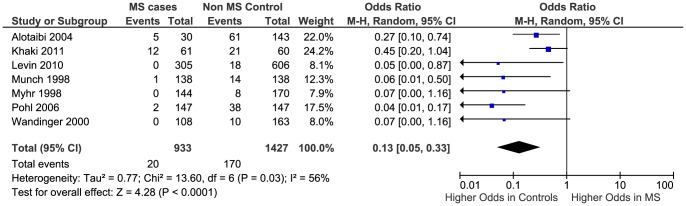
Forest plot of the Odds ratio for anti-EBV IgG sero-negativity.

The meta-analysis generated an overall OR of 0.13 (95% CI 0.05 to 0.33, p<0.0001) (Figure 8). Heterogeneity between studies was again significant I^2^ = 56% (p = 0.03). Due to the small number of studies in the analysis, a funnel plot was not used to assess the risk of publication bias.

### Subgroup analysis

To examine the effect of different factors on the overall OR for anti-EBV sero-positivity, we conducted eight subgroup analyses (Tables 2, 3, 4, 5, 6, 7, 8, and 9), five of which were *post hoc* (Tables 4, 5, 7, 8, and 9). Of note is that none of the subgroup differences reached statistical significance although there are some trends seen in the results.

**Table 2:** Combined OR in paediatric vs. adult studies.

Specificity of Antibody	Paediatric studies	Adult Studies	Difference significance (P value)
	OR (99% CI)	OR (99% CI)	
EBNA	5.48 (3.29–9.14)/7 studies	4.12 (2.39–7.10)/24 studies	0.32
VCA	5.72 (2.82–11.58)/6 studies	3.79 (1.62–8.82)/19 studies	0.34
EA	0.72 (0.1–5.29)/3 studies	1.51 (0.86–2.66)/11 studies	0.36

Note: P value is significant at or below 0.01.

**Table 3:** Combined OR in studies below median latitude vs. above the median.

Specificity of Antibody	Studies below median latitude (29.25–45. 37) North	Studies above median latitude (45.39–63.49) North	Difference significance (P value)
	OR (99% CI)	OR (99% CI)	
EBNA	3.81(1.86–7.83)/13 studies	6.32 (3.91–10.20)/16 studies	0.13
VCA	6.16 (3.67–10.35)/12 studies	2.99 (0.74–12.12)/10 studies	0.21
EA	1.45 (0.35, 6.08)/5 studies	1.33 (0.72, 2.46)/8 studies	0.88

Note: P value is significant at or below 0.01.

**Table 4:** Combined OR in studies matching for age vs. not matching for age.

Specificity of Antibody	Studies Matching for Age	Studies not Matching for age	Difference significance (P value)
	OR (99% CI)	OR (99% CI)	
EBNA	5.80 (3.13, 10.75)/20 studies	3.23 (2.09, 4.97)/11 studies	0.04
VCA	5.26 (2.20, 12.54)/16 studies	3.67 (1.69, 7.98)/9 studies	0.43
EA	1.52 (0.86, 2.69)/11 studies	0.77 (0.21, 2.79)/3 studies	0.21

Note: *post hoc* analyses. P value is significant at or below 0.01.

**Table 5:** Combined OR in studies matching for sex vs. not matching for sex.

Specificity of Antibody	Studies Matching for sex	Studies not Matching for sex	Difference significance (P value)
	OR (99% CI)	OR (99% CI)	
EBNA	5.52 (2.75, 11.06)/16 studies	3.64 (2.38, 5.56)/15 studies	0.19
VCA	6.79 (2.93, 15.70)/13 studies	3.03 (1.51, 6.09)/12 studies	0.06
EA	1.62 (0.83, 3.16)/8 studies	1.04 (0.40, 2.73)/6 studies	0.33

Note: *post hoc* analyses. P value is significant at or below 0.01.

**Table 6:** Combined OR in studies using McDonlad/Poser criteria vs. other/criteria not specified.

Specificity of Antibody	Studies using McDonlad/Poser Criteria	Studies using other criteria/criteria not specified	Difference significance (P value)
	OR (99% CI)	OR (99% CI)	
EBNA	4.14 (2.14, 7.99)/15 studies	4.86 (3.11, 7.58)/16 studies	0.6
VCA	4.42 (1.58, 12.38)/10 studies	4.58 (2.05, 10.20)/15 studies	0.9
EA	1.44 (0.66, 3.14)/7 studies	1.33 (0.69, 2.55)/8 studies	0.84

Note: P value is significant at or below 0.01.

**Table 7:** Combined OR in studies with confirmed MS cases vs. Confirmed and probable cases.

Specificity of Antibody	Confirmed MS	Confirmed and probable MS	Difference significance (P value)
	OR (99% CI)	OR (99% CI)	
EBNA	5.25 (3.37, 8.16)/18 studies	3.41 (1.69, 6.87)/13 studies	0.18
VCA	6.23 (2.96, 13.10)/14 studies	3.12 (1.30, 7.51)/11 studies	0.12
EA	1.33 (0.62, 2.83)/8 studies	1.53 (0.50, 4.66)/5 studies	0.07

Note: *post hoc* analyses. P value is significant at or below 0.01.

**Table 8:** Combined OR in studies using IFA vs. ELISA.

Specificity of Antibody	IFA	ELISA	Difference significance (P value)
	OR (99% CI)	OR (99% CI)	
EBNA	3.95 (2.04, 7.64)/8 studies	4.24 (2.46, 7.32)/21 studies	0.83
VCA	6.71 (2.40, 18.76)/12 studies	3.38 (1.50, 7.65)/13 studies	0.18
EA	2.24 (1.11, 4.52)/4 studies	1.08 (0.61, 1.92)/10 studies	0.04

Note: *post hoc* analyses. P value is significant at or below 0.01.

**Table 9:** Combined OR in studies with quality assessment score of 6 (median) or above vs. below 6.

Specificity of Antibody	Quality 6 and above	Quality less than 6	Difference significance (P value)
	OR (99% CI)	OR (99% CI)	
EBNA	5.24 (2.68, 10.23)/14 studies	3.59 (2.27, 5.67)/17 studies	0.23
VCA	5.21 (2.40, 11.29)/12 studies	3.89 (1.52, 9.97)/13 studies	0.54
EA	1.57 (0.86, 2.86)/8 studies	1.17 (0.40, 3.43)/6 studies	0.54

Note: *post hoc* analyses. P value is significant at or below 0.01.

Paediatric studies had a slightly higher OR than adult studies for anti-EBNA and anti-VCA sero-positivity (above 5 compared to 4 or below) (Table 2). Subgroup analysis by the latitude of studies did not reveal a clear trend, with a higher OR for anti-EBNA sero-positivity in studies above the median latitude (45.37 north) while the OR for anti-VCA was higher in studies below the median latitude (Table 3).

Studies that matched for age or sex had higher ORs for all anti-EBV IgG sero-positivities compared to those that did not (Tables 4 and 5). The ORs for all anti-EBV IgG sero-positivities was similar in studies that stated using McDonald/Poser criteria, compared to those that did not specify, or used different criteria (Table 6). However, when we compared studies with confirmed MS cases to those with confirmed and probable/CIS cases, the OR was higher in the former group for anti-EBNA and VCA sero-positivity (Table 7). Studies that used IFA as method of detection of anti-EBV IgG, had double the OR for anti-VCA and EA sero-positivity, but not for anti-EBNA, compared to studies that used ELISA (Table 8).

### Quality assessment

In our modified NOS scale the maximum score that could be achieved by a study was 12 stars. The majority of studies scored below half this with an overall median of five. The highest scoring studies were by Ascherio, *et al*., Levin, *et al*., and Ponsonby, *et al*., with score of 11 stars [18], [33], [42] (Table 10).

**Table 10:** Quality assessment of included studies.

Study ID	Selection				Comparability		Exposure					Total
	S1	S2	S3	S4	C1	C2	E1 a	E1 b	E2	E3	E4	
Alotaibi 2004[17]	*	–	–	*	*	–	**	*	–	*	*	8
Ascherio 2001[18]	*	*	*	*	*	*	**	–	*	*	*	11
Banwell 2007[19]	*	–	–	*	*	–	**	*	–	*	*	8
Bray 1983[20]	–	*	–	–	*	*	–	–	–	*	–	4
Buljevac 2005[21]	*	–	–	*	*	–	–	–	*	*	*	6
Comabella 2010[22]	–	*	–	*	*	–	–	–	–	*	–	4
Ferrante 1987[23]	–	–	–	–	–	*	–	–	–	*	–	2
Gutierrez 2002[24]	–	–	–	–	–	–	–	–	–	*	–	1
Haahr 2004[25]	–	–	–	*	*	*	–	–	–	*	–	4
Ingram 2010[26]	–	–	–	*	–	–	–	–	*	*	–	3
Jafari 2010[27]	*	–	–	*	–	–	–	–	–	*	–	3
Jaquiery 2010[28]	–	*	–	–	–	–	–	–	–	*	–	2
Jilek 2008[29]	*	–	–	–	–	–	–	–	*	*	–	3
Khaki 2011[30]	*	–	–	*	*	*	–	–	*	*	–	6
Lalive 2011[31]	–	–	–	*	–	–	–	–	–	*	–	2
Larsen 1985[32]	–	–	–	*	*	*	**	–	*	*	–	7
Levin 2010[33]	*	*	*	*	*	*	**	–	*	*	*	11
Lindsey 2010[34]	–	–	–	–	*	*	–	–	*	*	–	4
Lucas 2011[35]	*	*	*	*	*	*	–	–	*	*	*	9
Martyn 1993[36]	–	–	–	*	*	–	–	–	–	*	–	3
Mowry 2011[37]	–	–	–	*	–	–	**	–	*	*	–	5
Munch 1998[38]	–	–	–	*	*	*	–	–	–	*	–	4
Myhr 1998[39]	*	*	–	*	*	*	–	–	–	*	–	6
Nociti 2010[40]	*	–	–	*	–	–	–	–	*	*	–	4
Pohl 2006[41]	*	–	–	*	*	*	**	–	*	*	–	8
Ponsonby 2005[42]	*	*	*	*	*	*	**	*	*	*	–	11
Riverol 2007[43]	*	–	–	*	*	*	–	–	*	*	*	7
Sellner 2010[44]	*	–	–	*	*	–	–	–	*	*	–	5
Selter 2010[45]	–	*	–	*	–	–	–	–	*	*	–	4
Shirodaria 1987[46]	–	–	–	*	*	*	–	–	*	*	–	5
Sumaya 1980[47]	*	–	–	*	–	–	–	–	–	*	–	3
Sumaya 1985[48]	*	–	–	*	*	*	–	–	–	*	–	5
Sundqvist 2012[49]	*	*	*	*	*	*	–	–	*	*	–	8
Sundström 2004[50]	*	*	*	*	*	*	**	–	–	*	*	10
Villegas 2011[51]	*	–	–	*	–	–	–	–	–	*	–	3
Villoslada 2003[52]	*	–	–	*	*	*	–	–	*	*	–	6
Wandinger 2000[53]	*	*	–	*	*	*	–	–	*	*	–	7
Waubant 2011[54]	*	–	–	*	*	*	**	–	*	*	–	8
Zivadinov 2006[55]	*	*	–	*	*	*	–	–	–	*	–	6

**Abbreviations: C1**: Matching for age, **C2**: Matching for additional factor, **E1 a**: Blinding of sample analysts, **E1 b**: Conducted analysis in clinical laboratory, **E2**: Explicit serology cut-off values reported, **E3**: Same serology method used for cases and controls, **E4**: Missing data reported, **S1**: Adequate definition of cases, **S2**: Consecutive or obviously representative cases, **S3**: Adequate community controls, **S4**: Adequate definition of controls.

For selection criteria, the majority of studies had adequate definitions of cases and controls. However only a quarter of the studies recruited an obviously representative or a consecutive sample of cases, and only six studies recruited adequate community controls without an obvious source of bias. For the comparability criteria, 28 out of 39 studies matched cases and controls for at least one factor, with half of the studies matching for age and at least one additional factor. For the exposure criteria, only a quarter of studies reported blinding sample analysts, with three studies reporting conducting the serology in clinical laboratory (distant from the investigators). Half of the studies reported explicitly serological cut off values with only eight studies reporting whether there were missing data (related to participants' recruitment and withdrawal/analytical failures).

To examine the effect of the quality of studies on the OR for anti-EBV sero-positivity, we compared studies with a quality assessment score above the median (a score of five) with studies scoring at or below the median in a *post hoc* analysis. The OR of sero-positivity was higher for all anti-EBV IgG outcomes in studies scoring above the median (Table 9), although there was no statistically significant difference.

## Discussion

This review has again found an association between EBV sero-positivity, based on anti-EBNA and anti-VCA IgG testing. We also found no evidence for a difference in sero-prevalence of anti-EA IgG, indicative of recent infection.

There was considerable between study heterogeneity, and we examined different factors that might have been influential. Although none of the subgroup analyses reached statistical significance, several trends emerged. There was a slightly higher OR in paediatric studies compared to adult studies. This might be connected to higher overall exposure of adults to EBV infection than paediatric controls. However, Pakpoor *et al*. [8] found the OR of sero-negativity to anti-EBV antibodies to be similar in paediatric and adult MS cases. This discrepancy in the findings may be due to chance or the small number of paediatric studies included (only three) by Pakpoor, *et al*. [8]

We did not find a difference in combined OR between studies using more recent criteria (McDonald/Poser) compared to older criteria or failure to report criteria (which may simply reflect lack of reporting). The *post hoc* analysis according to the diagnosis status of MS participants revealed a higher combined OR in studies recruiting only confirmed vs. confirmed and probable/CIS patients for exposure to anti-EBNA and VCA IgG. This finding is plausible as unconfirmed MS patients are more likely to be misclassified and therefore have a lower prevalence of EBV exposure than true MS patients.

Studies matching for age and/or sex were found to have higher ORs for anti-EBNA and VCA IgG sero-positivity. Other important confounders are geographical location and socio-economic status. We could not test for the effect of controlling for these factors because few studies explicitly mentioned controlling for them. Comparison of studies according to their latitude did not reveal a trend. In some studies the exact area of recruitment was not mentioned and an estimate had to be used. A recent study by Disanto *et al*. [57] found that higher latitude was associated with higher prevalence of EBV exposure, independent from MS status.

Comparing the OR in studies that used IFA to detect anti-EBV antibodies vs. those that used ELISA, revealed that the former had double the OR for anti-VCA and anti-EA IgG sero-positivity but similar results for anti-EBNA. These results are consistent with the analysis by Pakpoor *et al*. [8] who found that studies that used IFA generated a much lower OR for sero-negativity of anti-EBV antibodies in MS cases compared studies which used ELISA.

Other factors that may contribute to the differences in estimates of ORs are the selection and source of cases and controls. Few studies clearly described the method of selection of cases (consecutive, random, all, or a percentage of the eligible cases). Ideally, the community would be the best source for controls [58]. Only six studies were deemed to have appropriate community controls [18], [33], [35], [42], [49], [50]. There were seven studies which recruited blood/bone marrow donors, which we considered inappropriate community controls, as such donors are selected for non-health risk behaviour. Looking at the results of six studies which recruited a control sample of patients with other neurological diseases (OND) [23], [24], [27], [28], [37], [40] and comparing them to seven studies which recruited only seemingly healthy hospital controls [17], [19], [26], [38], [39], [41], [51], we observed that, among the former group, only two out of the six studies found a statistically significant higher anti-EBV IgG sero-prevalence in cases [27], [37] compared to all seven studies in the latter group.

At least half of the studies scored poorly in our modified NOS scale for quality assessment. This may partly relate to poor reporting rather than poor conduct. This highlights the importance of following a standardised method for reporting to ensure that all relevant information is reported and to facilitate interpretation and validity assessment of studies. One such method is the STROBE statement (Strengthening the Reporting of Observational Studies in Epidemiology), which was developed to address standardised reporting of observational studies [59]. The NOS scale presently does not allow for uncertainty in quality assessment. Having the option to choose unclear in the criteria for assessment as used by the tool for assessing risk of bias in randomised controlled trials developed by the Cochrane Collaboration [13] would be helpful. Despite the limitation of the NOS quality assessment scale, we did find that studies with better methodological conduct are more likely to find stronger associations between EBV exposure and MS.

One major strength of this review is a more thorough search strategy with no language restriction. Despite this, we only found a single published study from areas other than Europe, North America and Australia (Asian study–Khaki *et al*. [30]). This emphasises the need for future studies to test the association of EBV with MS in other areas of the world with very different, generally lower prevalences of MS.

For consistent handling of data, we analysed the crude data in our meta-analyses, as very few studies provided adjusted odds ratios [18], [19], [54], nor adjusted for the same factors. We did not separate out prospective data presented in three studies [18], [33], [50] from the main meta-analyses, but removing these data has no effect on the results (data available from the authors). The funnel plot for anti-EBNA IgG sero-positivity revealed the possibility of publication bias, but this is unlikely to negate the main association.

Our review includes more than double the number of studies included in the most recently published systematic review by Santiago *et al*. [10]; the results are similar but more precise.

Although Santiago *et al*. [10] did assess the quality of included studies, they did not examine the potential for publication bias, and used more limited quality assessment. We found a similar result for sero-negativity to the systematic review by Pakpoor *et al*. [8] who did not undertake any quality assessment of the studies.

The possible role for EBV as a cause of MS provides the potential to prevent or reduce MS through vaccination against EBV [60]. Further research related to EBV and MS could focus on further characterising the risk of MS based on titres of anti-EBV antibodies. Although there have been studies which examined titre levels and MS risk [18], [50], [61], [62], the data have not yet been combined in a systematic review. Better characterisation of anti-EBV antibodies titres and the associated risk of MS could potentially identify high risk groups and help in prevention or management.

In conclusion, there is strong epidemiological evidence that MS patients have higher sero-prevalence of anti-EBV antibodies compared to controls.

## Supporting Information

Appendix S1 **MeSH/Emtree headings and text words in search strategy.**
(DOCX)

Checklist S1 **MOOSE Checklist.**
(DOCX)
