# Supplementary material for: Systematic Review and Meta-Analysis of the Sero-Epidemiological Association between Epstein Barr Virus and Multiple Sclerosis
Source: PLoS One. 2013 Apr 9;8(4):e61110. doi: 10.1371/journal.pone.0061110 (PMC3621759; doi:10.1371/journal.pone.0061110)
Supplement: Appendix S1 — MeSH/Emtree headings and text words in search strategy. (DOCX) [file pone.0061110.s001.docx]

**Appendix S1 - MeSH/ Emtree headings and text words in search strategy (MS terms combined with EBV terms)**

| Database | Multiple Sclerosis | Epstein Barr virus |
| --- | --- | --- |
| Medline MeSH Headings | 1. exp Demyelinating Autoimmune Diseases, CNS/ 2. Optic Neuritis/ | 1. Herpesvirus 4, Human/ 2. exp Epstein-Barr Virus Infections/ |
| Embase Emtree headings | 1. multiple sclerosis/ 2. demyelinating disease/ 3. Central Nervous system disease/ 4. myelooptic neuropathy/ 5. encephalomyelitis/ | 1. Epstein Barr virus/ |
| Text words (used in both databases) | 1. Multiple sclerosis.tw 2. disseminated sclerosis.tw 3. ms.tw 4. Neuromyelitis optica.tw or Optic neuritis.tw or devic.tw 5. transverse myelitis.tw 6. encephalomyelitis.tw 7. demyelinating disease.tw or inflammatory demyelinating.tw or Demyelinating Autoimmune Diseases.tw | 1. epstein barr.tw 2. EBV.tw 3. Mononucleosis.tw 4. glandular fever.tw 5. herpesvirus 4.tw or HHV 4.tw 6. kissing disease.tw |
