# Supplementary material for: Systematic Review and Meta-Analysis of the Sero-Epidemiological Association between Epstein Barr Virus and Multiple Sclerosis
Source: PLoS One. 2013 Apr 9;8(4):e61110. doi: 10.1371/journal.pone.0061110 (PMC3621759; doi:10.1371/journal.pone.0061110)
Supplement: Checklist S1 — MOOSE Checklist. (DOCX) [file pone.0061110.s002.docx]

**Meta-analysis of Observational Studies in Epidemiology (MOOSE) Checklist**

**Systematic review and meta-analysis of the sero-epidemiological association between Epstein Barr virus and Multiple sclerosis**

| **Criteria** | | **Brief description of how the criteria were handled in the meta-analysis** |
| --- | --- | --- |
| **Reporting of background should include** | |  |
| √ | Problem definition | A role for Epstein Barr virus (EBV) in the development of Multiple Sclerosis (MS) has been suggested by various sero-epidemiological studies that compared the prevalence of anti-EBV antibodies in serum of MS cases and controls, with an almost consistent finding of higher prevalence in MS patients. The last review that updated the association between MS and sero-positivity of different anti-EBV antibodies was the study of Santiago, et al., (2010). Many sero-epidemiological studies have been published since then and there is a need for a more up to date review of the evidence with a more inclusive search strategy. |
| √ | Hypothesis statement | MS cases are associated with higher prevalence of anti-EBV antibodies than controls |
| √ | Description of study outcomes | Prevalence of anti-EBV IgG antibodies in serum of MS cases compared to controls  Odds ratio (OR) of exposure to anti-EBV antibodies in MS cases compared to controls |
| √ | Type of exposure or intervention used | IgG antibodies against the following EBV antigens:  Epstein Barr Nuclear Antigen 1 (EBNA1)  Viral Capsid Antigen (VCA)  Early Antigen (EA) |
| √ | Type of study designs used | Case-control and nested cases control among cohort studies, that examined the prevalence of anti-EBV antibodies (IgG only) in serum of MS cases and non MS controls |
| √ | Study population | No restriction applied |
| **Reporting of search strategy should include** | |  |
| √ | Qualifications of searchers | The credentials of the three investigators (who contributed to the search strategy) YA, AA and CF are indicated in the authors list and the Acknowledgments section. |
| √ | Search strategy, including time period included in the synthesis and keywords | Medline from 1960 – March 2012  EMBASE from 1960 – March 2012  See Methodology and Appendix 1 |
| √ | Databases and registries searched | Medline and EMBASE |
| √ | Search software used, name and version, including special features | OvidSP, We detailed the MeSH/ Emtree Headings and text words in Appendix 1 |
| √ | Use of hand searching | Bibliographies of the retrieved papers (only the included studies) were hand searched for additional references, |
| √ | List of citations located and those excluded, including justifications | Details of the literature search process are outlined in the PRISMA flow chart. The citation list of excluded articles is available upon request |
| √ | Method of addressing articles published in languages other than English | We placed no restrictions on language; We were able to obtained all articles potentially eligible for inclusion in English language |
| √ | Method of handling abstracts and unpublished studies | We contacted a number of authors for full report of relevant unpublished studies |
| √ | Description of any contact with authors | We contacted authors of relevant articles for full report of their unpublished studies, or for extra data about anti-EBV antibodies prevalence in cases and controls. For details of the authors who provided extra unpublished information see the Acknowledgments section |
| **Reporting of methods should include** | |  |
| √ | Description of relevance or appropriateness of studies assembled for assessing the hypothesis to be tested | Detailed inclusion and exclusion criteria are described in the paper |
| √ | Rationale for the selection and coding of data | A data extraction sheet was developed (available on request). Data extracted were related to bibliographic details of included study, method of identification of the study, Characteristics of cases/ controls, Outcomes and quality assessment |
| √ | Assessment of confounding | We conducted eight subgroup analyses to compare the effects of a number of potential confounders on the OR of exposure to anti-EBV antibodies. |
| √ | Assessment of study quality, including blinding of quality assessors; stratification or regression on possible predictors of study results | We used a modified version of the Newcastle Ottawa Scale (NOS) to assess the quality of each study. We conducted a subgroup analysis comparing the studies which scored above the median in the NOS scale to those scoring below the median |
| √ | Assessment of heterogeneity | We used the I^2^ value to assess heterogeneity |
| √ | Description of statistical methods in sufficient detail to be replicated | We mentioned type of analysis we used (meta-analysis and subgroup meta-analysis) and type of software we used (Review Manager 5) |
| √ | Provision of appropriate tables and graphics | We included PRISMA flow chart to show the method of studies identification, Table1 showing characteristics of included studies, Table 2 – Table 9 showing Results of Subgroup analysis, Table 10 showing results of Quality assessment, Four forest plots of the main different meta-analyses conducted and Three Funnel plots for publication bias assessment. |
| **Reporting of results should include** | |  |
| √ | Graph summarizing individual study estimates and overall estimate | Figure 2, Figure 4, Figure 6 and Figure 8 |
| √ | Table giving descriptive information for each study included | Table 1 |
| √ | Results of sensitivity testing | Table 2 – Table 9 |
| √ | Indication of statistical uncertainty of findings | 95% and 99% CI intervals were presented for all analyses together with I^2^ values for the three main meta-analyses |
| **Reporting of discussion should include** | |  |
| √ | Quantitative assessment of bias | Results of subgroup analyses are discussed with main potential confounding factors discussed. Results of Funnel plot and risk of publication bias is highlighted |
| √ | Justification for exclusion | Reasons for exclusion were reported mainly in the results section, with the two main reasons being either that studies were irrelevant (Did not measure outcome of interest) or did not have clear information with no extra data obtainable. Likelihood of publication bias was discussed. |
| √ | Assessment of quality of included studies | Implication of the subgroup analyses related to quality assessment was highlighted |
| **Reporting of conclusions should include** | |  |
| √ | Consideration of alternative explanations for observed results | We mentioned in the discussion that included patients with unconfirmed MS diagnosis (clinically isolated syndrome) could have altered the results |
| √ | Generalization of the conclusions | We reported the fact that almost all of the studies were from Europe, North America and Australia. We highlighted that there few/ none found from Asia and Africa |
| √ | Guidelines for future research | Further research related to EBV and MS could focus on characterising the risk of MS based on titres of anti-EBV antibodies. Although there have been studies published which examined titre levels and MS risk, a meta-analysis combining data from different studies is yet to be conducted |
| √ | Disclosure of funding source | No funding was required for conducting this review |
